# Supplementary material for: Is there a link between endowment inequality and deception? – an analysis of students and chess players
Source: PLoS One. 2022 Jan 27;17(1):e0262144. doi: 10.1371/journal.pone.0262144 (PMC8794128; doi:10.1371/journal.pone.0262144)
Supplement: S1 File — (PDF) [file pone.0262144.s001.pdf]

[Logo of the university]

Martin Luther University
Halle-Wittenberg

A warm welcome to our scientific study!

As part of a research project at the Martin Luther University Halle-Wittenberg, we want to better understand human decision-making behavior. To be more precisely, we aim at examining the communication behavior under different conditions. Therefore we are conducting a study, which consists an experiment and a short questionnaire. In order to make the situation more realistic, 20% of the participants are randomly selected and are paid in line with decisions made in the experiment. In addition, 10 randomly selected participants will receive a show-up fee of €50. To enable us to contact you in case of success, we ask you to provide your e-mail address at the end of the survey. It will be deleted after the prize money has been paid out.

To attend the study, you must be at least 18 years old. In total, participation in the study will take about 8-10 minutes of your time. Participation is of course voluntary. The collected data will be treated strictly confidential. It is not possible to draw conclusions about individuals.

Thank you very much for your participation. If you have any questions or feedback, please feel free to contact:

- Sven Grüner (Martin Luther University Halle-Wittenberg)
  sven.gruener@landw.uni-halle.de
- Ilia Khassine
  iliakhassine@gmail.com

I have read the above information and agree to participate in this research project. ☐

First of all we have some general questions about you

A) Are you enrolled as a student at a university?

○ Yes (=1) ○ No (=0)

B) Do you actively play chess in a club?

○ Yes (=1) ○ No (=0)

C) In which federal state do you live (main residence)?

Saxony-Anhalt (1), Saxony (2), Thuringia (3), Mecklenburg Western Pomerania (4), Brandenburg (5), Berlin (6), Bavaria (7), Bremen (8), Hesse (9), Hamburg (10), Baden-Württemberg (11), Lower Saxony (12), Northrhine-Westphalia (13), Rhineland Palatinate (14), Saarland (15), Schleswig Holstein (16)

Subjects are randomly assigned to 1 of 6 version

Experimental Instructions [1A]

Description of the experiment

You participate in a 2-person experiment. You will find two options A and B. These include monetary consequences for you and a randomly selected opponent. Neither you nor your opponent will receive more detailed information about the identity of the other person. Your task in the experiment is to send a message to the opponent which option results in a higher payoff for her. There are two messages to choose from: Message 1 (Option A makes you earn more money than Option B) *or* Message 2 (Option B makes you earn more money than Option A). The message can be true or false. Your opponent has less information. She does not know the monetary payoffs of the two options. Your opponent only knows the message you sent. However, your opponent's decision is relevant for the payoff, i.e., the option chosen by your opponent determines the monetary consequences for you and him.

Example

This can be better illustrated with an example. Let us look at the following decision situation.

Decision situation

The options and messages read as follows:

**Options**	**Message to the opponent**
A: €1 for you and €2 for the opponent	1: Option A makes you earn more money than option B.
B: €3 for you and €4 for the opponent	2: Option B makes you earn more money than option A.

- Your task is to send a message to your opponent. You can choose between message 1 ("Option A will make you earn more money than Option B.") and message 2 ("Option B will make you earn more money than Option A.").
- Your randomly selected opponent will only be informed about your message. The opponent decides whether or not to follow your message. The opponent's decision determines which option is actually played.
- If option A is played, you receive €1 and your opponent receives €2. If Option B is played, you receive €3 and your opponent receives €4.

How does the payment process work?

The sums of money represent real amounts of money. In total you will see 2 independent decision situations. In the experiment, your decisions and the decisions of a randomly selected opponent are matched. Among those who complete the form, 20% of the participants are randomly selected and the decisions one randomly selected decision situation are paid out (i.e. you receive money). In case of success you will be notified in a private email.

Now it is your turn to make decisions.

Your decision 1

As a reminder

- In contrast to your opponent, you know the exact monetary consequences of both options A and B.
- Your opponent only knows the message (1 or 2) that you send to her.
- Your opponent chooses one option (A or B), which then becomes payable for both players.

Decision situation 1

The options and messages read as follows:

**Options**	**Message to the opponent**
A: 9€ for you and €12 for the opponent	1: Option A makes you earn more money than option B.
B: 10€ for you and €3 for the opponent	2: Option B makes you earn more money than option A.

Please fill out the following field:

Decision: I send message **……………………………………**(Please enter: 1 or 2)

Your decision 2

As a reminder

- In contrast to your opponent, you know the exact monetary consequences of both options A and B.
- Your opponent only knows the message (1 or 2) that you send to her.
- Your opponent chooses one option (A or B), which then becomes payable for both players.

Decision situation 2

The options and messages read as follows:

**Options**	**Message to the opponent**
A: €6 for you and €15 for the opponent	1: Option A makes you earn more money than option B.
B: €5 for you and €5 for the opponent	2: Option B makes you earn more money than option A.

Please fill out the following field:

Decision: I send message **……………………………………**(Please enter: 1 or 2)

Expectations

Reminder

- In contrast to your opponent, you know the exact monetary consequences of the options A and B.
- Your opponent only knows the message (1 or 2) you send her.
- Your opponent chooses one option (A or B), which then becomes payable for both players.

What expectations did you have when you made your decisions?

Expectation: I suppose that **……………………….**[Please enter a number from 0 to 100] percent of the opponents will follow my message (i.e., will opt for the option I sent).

Experimental Instructions [2A]

Description of the experiment

You participate in a 2-person experiment. You will find two options A and B. These include monetary consequences for you and a randomly selected opponent. Neither you nor your opponent will receive more detailed information about the identity of the other person. Your task in the experiment is to send a message to the opponent which option results in a higher payoff for her. There are two messages to choose from: Message 1 (Option A makes you earn more money than Option B) *or* Message 2 (Option B makes you earn more money than Option A). The message can be true or false. Your opponent has less information. She does not know the monetary payoffs of the two options. Your opponent only knows the message you sent and differences in initial wealth. However, your opponent's decision is relevant for the payoff, i.e., the option chosen by your opponent determines the monetary consequences for you and him.

Example

This can be better illustrated with an example. Let us look at the following decision situation.

Decision situation

Regardless of your decision, the following situation exists with respect to the initial wealth, which is known to both your opponent and yourself: Your initial wealth is €10. Your opponent's initial wealth is €0.

The options and messages read as follows:

**Options**	**Message to the opponent**
A: €1 for you and €2 for the opponent	1: Option A makes you earn more money than option B.
B: €3 for you and €4 for the opponent	2: Option B makes you earn more money than option A.

- Your task is to send a message to your opponent. You can choose between message 1 ("Option A will make you earn more money than Option B.") and message 2 ("Option B will make you earn more money than Option A.").
- Your randomly selected opponent will only be informed about your message and the initial wealth. The opponent decides whether or not to follow your message. The opponent's decision determines which option is actually played.
- If option A is played, you receive €1 (in addition to the initial wealth of €10) and your opponent receives €2. If Option B is played, you receive €3 (in addition to the initial wealth of €10) and your opponent receives €4.

How does the payment process work?

The sums of money represent real amounts of money. In total you will see 2 independent decision situations. In the experiment, your decisions and the decisions of a randomly selected opponent are matched. Among those who complete the form, 20% of the participants are randomly selected and the decisions one randomly selected decision situation are paid out (i.e. you receive money). In case of success you will be notified in a private email.

Now it is your turn to make decisions.

Your decision 1

As a reminder

- In contrast to your opponent, you know the exact monetary consequences of both options A and B.
- Your opponent only knows the initial wealth of both players and the message (1 or 2) that you send to her.
- Your opponent chooses one option (A or B), which then becomes payable for both players.

Decision situation 1

Regardless of your decision, the following situation exists with respect to the initial wealth, which is known to both your opponent and yourself: Your initial wealth is €10. Your opponent's initial wealth is €0.

The options and messages read as follows:

**Options**	**Message to the opponent**
A: €9 for you and €12 for the opponent	1: Option A makes you earn more money than option B.
B: €10 for you and €3 for the opponent	2: Option B makes you earn more money than option A.

Please fill out the following field:

Decision: I send message **……………………………………**(Please enter: 1 or 2)

Your decision 2

As a reminder

- In contrast to your opponent, you know the exact monetary consequences of both options A and B.
- Your opponent only knows the initial wealth of both players and the message (1 or 2) that you send to her.
- Your opponent chooses one option (A or B), which then becomes payable for both players.

Decision situation 2

Regardless of your decision, the following situation exists with respect to the initial wealth, which is known to both your opponent and yourself: Your initial wealth is €10. Your opponent's initial wealth is €0.

The options and messages read as follows:

**Options**	**Message to the opponent**
A: €6 for you and €15 for the opponent	1: Option A makes you earn more money than option B.
B: €5 for you and €5 for the opponent	2: Option B makes you earn more money than option A.

Please fill out the following field:

Decision: I send message **……………………………………**(Please enter: 1 or 2)

Expectations

<u>Reminder</u>

- In contrast to your opponent, you know the exact monetary consequences of the options A and B.
- Your opponent only knows the initial wealth of both players and the message (1 or 2) you send her.
- Your opponent chooses one option (A or B), which then becomes payable for both players.
- **Regardless of your decision, the following situation exists with respect to the initial wealth, which is known to both your opponent and yourself: Your initial wealth is €10. Your opponent's initial wealth is €0.**

<u>What expectations did you have when you made your decisions?</u>

Expectation: I suppose that **……………………….**[Please enter a number from 0 to 100] percent of the opponents will follow <u>my message</u> (i.e., will opt for the option I sent).

Experimental Instructions [3A]

Description of the experiment

You participate in a 2-person experiment. You will find two options A and B. These include monetary consequences for you and a randomly selected opponent. Neither you nor your opponent will receive more detailed information about the identity of the other person. Your task in the experiment is to send a message to the opponent which option results in a higher payoff for her. There are two messages to choose from: Message 1 (Option A makes you earn more money than Option B) *or* Message 2 (Option B makes you earn more money than Option A). The message can be true or false. Your opponent has less information. She does not know the monetary payoffs of the two options. Your opponent only knows the message you sent and differences in initial wealth. However, your opponent's decision is relevant for the payoff, i.e., the option chosen by your opponent determines the monetary consequences for you and him.

Example

This can be better illustrated with an example. Let us look at the following decision situation.

Decision situation

Regardless of your decision, the following situation exists with respect to the initial wealth, which is known to both your opponent and yourself: Your initial wealth is €0. Your opponent's initial wealth is €10.

The options and messages read as follows:

**Options**	**Message to the opponent**
A: €1 for you and €2 for the opponent	1: Option A makes you earn more money than option B.
B: €3 for you and €4 for the opponent	2: Option B makes you earn more money than option A.

- Your task is to send a message to your opponent. You can choose between message 1 ("Option A will make you earn more money than Option B.") and message 2 ("Option B will make you earn more money than Option A.").
- Your randomly selected opponent will only be informed about your message and the initial wealth. The opponent decides whether or not to follow your message. The opponent's decision determines which option is actually played.
- If option A is played, you receive €1 and your opponent receives €2 (in addition to the initial wealth of €10). If Option B is played, you receive €3 and your opponent receives €4 (in addition to the initial wealth of €10).

How does the payment process work?

The sums of money represent real amounts of money. In total you will see 2 independent decision situations. In the experiment, your decisions and the decisions of a randomly selected opponent are matched. Among those who complete the form, 20% of the participants are randomly selected and the decisions one randomly selected decision situation are paid out (i.e. you receive money). In case of success you will be notified in a private email.

Now it is your turn to make decisions.

Your decision 1

As a reminder

- In contrast to your opponent, you know the exact monetary consequences of both options A and B.
- Your opponent only knows the initial wealth of both players and the message (1 or 2) that you send to her.
- Your opponent chooses one option (A or B), which then becomes payable for both players.

Decision situation 1

Regardless of your decision, the following situation exists with respect to the initial wealth, which is known to both your opponent and yourself: Your initial wealth is €0. Your opponent's initial wealth is €10.

The options and messages read as follows:

**Options**	**Message to the opponent**
A: €9 for you and €12 for the opponent	1: Option A makes you earn more money than option B.
B: €10 for you and €3 for the opponent	2: Option B makes you earn more money than option A.

Please fill out the following field:

Decision: I send message **……………………………………**(Please enter: 1 or 2)

Your decision 2

As a reminder

- In contrast to your opponent, you know the exact monetary consequences of both options A and B.
- Your opponent only knows the initial wealth of both players and the message (1 or 2) that you send to her.
- Your opponent chooses one option (A or B), which then becomes payable for both players.

Decision situation 2

Regardless of your decision, the following situation exists with respect to the initial wealth, which is known to both your opponent and yourself: Your initial wealth is €0. Your opponent's initial wealth is €10.

The options and messages read as follows:

**Options**	**Message to the opponent**
A: €6 for you and €15 for the opponent	1: Option A makes you earn more money than option B.
B: €5 for you and €5 for the opponent	2: Option B makes you earn more money than option A.

Please fill out the following field:

Decision: I send message **……………………………………**(Please enter: 1 or 2)

Expectations

Reminder

- In contrast to your opponent, you know the exact monetary consequences of the options A and B.
- Your opponent only knows the initial wealth of both players and the message (1 or 2) you send her.
- Your opponent chooses one option (A or B), which then becomes payable for both players.
- **Regardless of your decision, the following situation exists with respect to the initial wealth, which is known to both your opponent and yourself: Your initial wealth is €0. Your opponent's initial wealth is €10.**

What expectations did you have when you made your decisions?

Expectation: I suppose that **……………………….**[Please enter a number from 0 to 100] percent of the opponents will follow my message (i.e., will opt for the option I sent).

Experimental instructions [4b]

Description of the experiment

You will participate in a 2-person experiment. This involves 2 options (A and B), each of which contains monetary payoffs for you and your opponent. Your opponent knows the exact payoffs (i.e., exact amounts).

You only know the message that your opponent sends you. This message can be true or false. The two possible messages are:

Message 1: Option A makes you earn more money than option B.

Message 2: Option B makes you earn more money than option A.

You decide whether or not to follow the message sent by the other player. Your decision determines the payoffs for you and your opponent, which is common knowledge to you and your opponent.

What is the payment procedure?

In the experiment, your decisions and the decisions of a randomly selected opponent are matched. Neither you nor your opponent will receive more precise information from each other. The amounts of money mentioned represents real cash outs. Among those who complete the form, 20% of the participants are randomly selected and their decisions are paid out (i.e., you receive money). In case of success you will be notified in a private email.

Now it is your turn to make decisions.

Your decision

As a reminder

- Your opponent knows the exact monetary consequences of options A and B
- Your opponent knows that your only have information about the message sent to you.
- Your opponent knows that your decision (whether or not to follow the message) determines the payoffs of you and your opponent.

Your opponent sends you a message ("Option A makes you earn more money than Option B." or "Option B makes you earn more money than Option A.").

What is your decision? Do you follow the other player's message or do you decide otherwise?

**Decision**	Yes, I follow the message. ○1<br>No, I do not follow the message. ○0
**Expectation**	How many of 100 participants do you think sent you an <u>honest message</u>? ……………………… [Please insert a number from 0 to 100]

Experimental instructions [5b]

Description of the experiment

You will participate in a 2-person experiment. This involves 2 options (A and B), each of which contains monetary payoffs for you and your opponent. Your opponent knows the exact payoffs (i.e., exact amounts).

You only know the message that your opponent sends you. This message can be true or false. The two possible messages are:

Message 1: Option A makes you earn more money than option B.

Message 2: Option B makes you earn more money than option A.

You decide whether or not to follow the message sent by the other player. Your decision determines the payoffs for you and your opponent, which is common knowledge to you and your opponent.

Regardless of your decision, the following situation exists with respect to the initial wealth, which is known to both your opponent and yourself: Your initial wealth is €0. Your opponent's initial wealth is €10.

What is the payment procedure?

In the experiment, your decisions and the decisions of a randomly selected opponent are matched. Neither you nor your opponent will receive more precise information from each other. The amounts of money mentioned represents real cash outs. Among those who complete the form, 20% of the participants are randomly selected and their decisions are paid out (i.e., you receive money). In case of success you will be notified in a private email.

Now it is your turn to make decisions.

Your decision

<u>As a reminder</u>

- Your opponent knows the exact monetary consequences of options A and B, as well as the initial wealth of both players.
- Your opponent knows that you only have information about the initial wealth of both players and the message sent to you.
- Your opponent knows that your decision (whether or not to follow the message) determines the payoffs of you and your opponent.

Your opponent sends you a message ("Option A makes you earn more money than Option B." or "Option B makes you earn more money than Option A.").

What is your decision? Do you follow the other player's message or do you decide otherwise?

Regardless of your decision, the following situation exists with respect to the initial wealth, which is known to both your opponent and yourself: Your initial wealth is €0. Your opponent's initial wealth is €10.

**Decision**	Yes, I follow the message. ○1<br>No, I do not follow the message. ○0
**Expectation**	How many of 100 participants do you think sent you an <u>honest message</u>? ……………………… [Please insert a number from 0 to 100]

Experimental instructions [6b]

Description of the experiment

You will participate in a 2-person experiment. This involves 2 options (A and B), each of which contains monetary payoffs for you and your opponent. Your opponent knows the exact payoffs (i.e., exact amounts).

You only know the message that your opponent sends you. This message can be true or false. The two possible messages are:

Message 1: Option A makes you earn more money than option B.

Message 2: Option B makes you earn more money than option A.

You decide whether or not to follow the message sent by the other player. Your decision determines the payoffs for you and your opponent, which is common knowledge to you and your opponent.

Regardless of your decision, the following situation exists with respect to the initial wealth, which is known to both your opponent and yourself: Your initial wealth is €10. Your opponent‘s initial wealth is €0.

What is the payment procedure?

In the experiment, your decisions and the decisions of a randomly selected opponent are matched. Neither you nor your opponent will receive more precise information from each other. The amounts of money mentioned represents real cash outs. Among those who complete the form, 20% of the participants are randomly selected and their decisions are paid out (i.e., you receive money). In case of success you will be notified in a private email.

Now it is your turn to make decisions.

Your decision

<u>As a reminder</u>

- Your opponent knows the exact monetary consequences of options A and B, as well as the initial wealth of both players.
- Your opponent knows that you only have information about the initial wealth of both players and the message sent to you.
- Your opponent knows that your decision (whether or not to follow the message) determines the payoffs of you and your opponent.

Your opponent sends you a message ("Option A makes you earn more money than Option B." or "Option B makes you earn more money than Option A.").

What is your decision? Do you follow the other player's message or do you decide otherwise?

Regardless of your decision, the following situation exists with respect to the initial wealth, which is known to both your opponent and yourself: Your initial wealth is €10. Your opponent's initial wealth is €0.

**Decision**	Yes, I follow the message. ○1<br>No, I do not follow the message. ○0
**Expectation**	How many of 100 participants do you think sent you an <u>honest message</u>? ……………………… [Please insert a number from 0 to 100]

The remaining questions had to be answered by all participants.

We will now ask you now to answer a few questions about yourself.

1. In which degree program are you enrolled?

1O Agricultural sciences	5O Law	9O Psychology
2O Business economics	6O Mathematics	10O Sociology
3O History	7O Physics	11O Theology
4O Computer science	8O Political science	12O *Other field of study:* -------------------------

Note: If you are studying to become a teacher, please indicate the combination of subjects.

2. How old are you?

……………………. years

3. How many years have you been playing chess in a club?

……………….. years

4. What is your gender?

Male<br>=0	Female<br>=1	Other<br>=2
O	O	O

5. The next questions are about your attitude towards other people. Please indicate to what extent you agree with each statement.

	don't agree at all<br>=1	agree a bit<br>=2	agree somewhat<br>=3	agree mostly<br>=4	agree completely<br>=5	
I am convinced that most people have good intentions.	O	O	O	O	O	1
You can't rely on anyone these days.	O	O	O	O	O	2
In general, people can be trusted.	O	O	O	O	O	3

6. **In politics people often talk about "left" and "right" to distinguish different attitudes. If you think about your own political views: Where would you place them?**

   Please answer using the following scale. 0 means "entirely left", 10 means "entirely right". You can weigh your answers using the steps between 0 and 10.

0	entirely left	○
1		○
2		○
3		○
4		○
5		○
6		○
7		○
8		○
9		○
10	entirely right	○

7. **Now it's about your years of education. Please add up the years of school education, training, and university education (if applicable). How many years do you have?**

   …………………… years

8. **Do you belong to a church or religious group?**

Yes =1	No =0
○	○

9. People react to unfair situations in very different ways. In the following I would like to ask <u>you</u> how you would react in unfair situations. I will now read you several statements. Please tell me to what extent each of these statements apply to you. If you have never experienced such a situation yourself, try to imagine how you would react if you were in such a situation.

First, we will look at situations to the advantage of others and to your **<u>own disadvantage</u>**.

	not at all =1	=2	=3	=4	=5	exactly =6	
It makes me angry when others are undeservingly better off than me.	○	○	○	○	○	○	1
It worries me when I have to work hard for things that come easily to others.	○	○	○	○	○	○	2

Now, we will look at situations that turn out **<u>to your advantage</u>** and to the disadvantage of others.

	not at all =1	=2	=3	=4	=5	exactly =6	
I feel guilty when I am better off than others for no reason.	○	○	○	○	○	○	3
It bothers me when things come easily to me that others have to work hard for.	○	○	○	○	○	○	4

10. Is your net income

less than €750	1
€750 up to less than €1500	2
€1500 up to less than €2000	3
€2000 up to less than €2500	4
€2500 up to less than €3000	5
more than €3000	6

If you would like to participate in the lottery, please enter your e-mail address. So that we can contact you in case of success. Afterwards we will delete any personal data.

E-Mail: ______________________________________

Many thanks for your participation! In case you win a prize, we will contact you by e-mail after completion of the experimental procedure.
