# Supplementary material for: Is there a link between endowment inequality and deception? – an analysis of students and chess players
Source: PLoS One. 2022 Jan 27;17(1):e0262144. doi: 10.1371/journal.pone.0262144 (PMC8794128; doi:10.1371/journal.pone.0262144)
Supplement: S1 Appendix — (DOCX) [file pone.0262144.s002.docx]

# Appendix

# Further correlation analysis

## Correlation of expectations

The following table depicts how strongly various expectations are correlated with each other according to Spearman’s rho. For example, line 1 is about the correlation between students in the baseline scenario and students in treatment 1.

**Table A1 Correlation of expectations ^(a)^**

|  | **Expectations (H0: both are independent)** | **Sender behavior (Player 1)** | **Receiver behavior (Player 2)** |
| --- | --- | --- | --- |
| 1 | Students Baseline &  Students treatment 1 | -0.1612  (0.2184) | -0.2734  (0.0345) |
| 2 | Students Baseline &  Students treatment 2 | -0.1115  (0.3965) | -0.0803  (0.5419) |
| 3 | Non-Students Baseline &  Students treatment 1 | -0.2934  (0.0229) | 0.0649  (0.6222) |
| 4 | Non-Students Baseline &  Students treatment 2 | 0.0059  (0.9645) | -0.1689  (0.1971) |
| 5 | Students Baseline &  Non-Students Baseline | 0.1995  (0.1264) | -0.1168  (0.3741) |
| 6 | Students treatment 1 &  Non-Students treatment 1 | -0.0138  (0.9166) | 0.2589  (0.0458) |
| 7 | Students treatment 2 &  Non-Students treatment 2 | 0.2886  (0.0253) | -0.1921  (0.1414) |

(a) Note: Spearman’s rho and its respective p-value in brackets.

## Association between decisions and expectations (point-biserial correlation coefficient, pbis)

**Table A2 Correlation of decisions and expectations: player 1 (sender behavior) ^(a)^**

| **Situation** | **Scenario** | **Population** | **Coefficient** | **P-Value** | **df** |
| --- | --- | --- | --- | --- | --- |
| 1 | All | all | -0.0227 | 0.7627 | 178 |
| 1 | All | student | -0.0601 | 0.5738 | 88 |
| 1 | All | non-student | 0.0244 | 0.8194 | 88 |
| 1 | baseline | student | -0.2884 | 0.1223 | 28 |
| 1 | baseline | non-student | 0.1097 | 0.5637 | 28 |
| 1 | treatment 1 | student | 0.1611 | 0.3951 | 28 |
| 1 | treatment 1 | non-student | -0.0875 | 0.6455 | 28 |
| 1 | treatment 2 | student | 0.0099 | 0.9585 | 28 |
| 1 | treatment 2 | non-student | -0.0311 | 0.8705 | 28 |
| 2 | All | all | 0.1918 | 0.0099 | 178 |
| 2 | All | student | 0.2013 | 0.0571 | 88 |
| 2 | All | non-student | 0.1709 | 0.1072 | 88 |
| 2 | baseline | student | -0.2120 | 0.2608 | 28 |
| 2 | baseline | non-student | 0.0514 | 0.7874 | 28 |
| 2 | treatment 1 | student | 0.4000 | 0.0285 | 28 |
| 2 | treatment 1 | non-student | 0.4593 | 0.0107 | 28 |
| 2 | treatment 2 | student | 0.3669 | 0.0461 | 28 |
| 2 | treatment 2 | non-student | -0.1921 | 0.3092 | 28 |

(a) Note: Decision means sending an honest message.

**Table A3 Correlation of decisions and expectations: player 2 (receiver behavior) ^(a)^**

| **Situation** | **Scenario** | **Population** | **Coefficient** | **P-Value** | **df** |
| --- | --- | --- | --- | --- | --- |
| 1 | All | all | 0.5739 | 0.0001 | 178 |
| 1 | All | student | 0.5199 | 0.0001 | 88 |
| 1 | All | non-student | 0.6167 | 0.0001 | 88 |
| 1 | baseline | student | 0.3933 | 0.0315 | 28 |
| 1 | baseline | non-student | 0.4083 | 0.0251 | 28 |
| 1 | treatment 1 | student | 0.6549 | 0.0001 | 28 |
| 1 | treatment 1 | non-student | 0.7091 | 0.0001 | 28 |
| 1 | treatment 2 | student | 0.5405 | 0.0020 | 28 |
| 1 | treatment 2 | non-student | 0.6988 | 0.0001 | 28 |

(a) Note: Decision means to trust the message of player 1 (more precisely, following the message of player 1).

## Cramer’s V

**Table A4 Cramer’s V (sender behavior)**

| **Population** | **Situation** | **Comparison** | **V** | **p-value** |
| --- | --- | --- | --- | --- |
| Student | 1 | baseline vs T1 | 0.2334 | 0.071 |
| Student | 1 | baseline vs T2 | -0.1110 | 0.390 |
| Non-student | 1 | baseline vs T1 | -0.1508 | 0.243 |
| Non-student | 1 | baseline vs T2 | -0.0405 | 0.754 |
| Student & Non-student | 1 | baseline | -0.0405 | 0.754 |
| Student & Non-student | 1 | T1 | 0.3333 | 0.010 |
| Student & Non-student | 1 | T2 | -0.1110 | 0.390 |
| Student | 2 | baseline vs T1 | 0.0000 | 1.000 |
| Student | 2 | baseline vs T2 | -0.0754 | 0.559 |
| Non-student | 2 | baseline vs T1 | -0.2023 | 0.117 |
| Non-student | 2 | baseline vs T2 | -0.0894 | 0.488 |
| Student & Non-student | 2 | baseline | -0.1292 | 0.317 |
| Student & Non-student | 2 | T1 | 0.0754 | 0.559 |
| Student & Non-student | 2 | T2 | -0.1155 | 0.371 |

**Table A5 Cramer’s V (receiver behavior)**

| **Population** | **Comparison** | **V** | **p-value** |
| --- | --- | --- | --- |
| Student | baseline vs T1 | -0.0788 | 0.542 |
| Student | baseline vs T2 | -0.2182 | 0.091 |
| Non-student | baseline vs T1 | 0.0358 | 0.781 |
| Non-student | baseline vs T2 | -0.1361 | 0.292 |
| Student & Non-student | baseline | -0.1508 | 0.243 |
| Student & Non-student | T1 | -0.0370 | 0.774 |
| Student & Non-student | T2 | -0.0673 | 0.602 |

# Further regressions to explain honest and trust behaviors

**Table A6 Regressions to explain honest behaviors**

| **Logit (Marginal effects)**  **Y=1, message honest**  **Y=0, else** | Situation 1 | | | | Situation 2 | | | |
| --- | --- | --- | --- | --- | --- | --- | --- | --- |
|  | I | II | III | IV | V | VI | VII | VIII |
| Non-student | -0.147850  (0.21629) | 0.007119 (0.10823) | 0.138354  (0.60009) | -0.10387 (0.59723) | 0.0659452 (0.20221) | 0.128813 (0.12113) | 0.9481851 (0.11867) | 0.949929  (0.11671) |
| Treatment 1 |  | 0.186292  (0.10812) | 0.144791  (0.09956) | 0.132850  (0.09626) |  | 0.00000  (0.10871) | 0.052472  (0.10195) | 0.054635  (0.10019) |
| Treatment 1 ∙ Non-student |  | -0.41496  (0.23186) | -0.369537  (0.26826) | -0.33573  (0.26785) |  | -0.210998  (0.20593) | -0.294206  (0.22636) | -0.23021  (0.22624) |
| Treatment 2 |  | -0.11530  (0.1118) | -0.156285  (0.1306) | -0.16581  (0.12934) |  | -0.054118  (0.10961) | -0.066747  (0.12327) | -0.06360  (0.12315) |
| Treatment 2 ∙ Non-student |  | 0.069416  (0.12073) | 0.101858  (0.08639) | 0.105284  (0.07826) |  | -0.034351  (0.17334) | -0.020394  (0.17647) | -0.00574  (0.16892) |
| Expectation | -0.001013  (0.00241) |  |  | -0.00278  (0.00226) | 0.004066 (0.00221) |  |  | 0.003594  (0.00237) |
| Expectation ∙ Non-student | 0.0015  (0.00319) |  |  | 0.003321  (0.00278) | -0.000587  (0.00312) |  |  | -0.00067  (0.00328) |
| Age |  |  | -0.021377  (0.01592) | -0.02354  (0.01561) |  |  | 0.034172  (0.0202) | 0.031513  (0.02036) |
| Age ∙ Non-student |  |  | 0.0158728  (0.01627) | 0.018115  (0.01599) |  |  | -0.038696  (0.02048) | -0.03707  (0.02066) |
| Female |  |  | 0.1223935  (0.08851) | 0.131460  (0.08196) |  |  | -0.077437  (0.12396) | -0.10669  (0.13002) |
| Female ∙ Non-student |  |  | -0.169772  (0.26444) | -0.19630  (0.27061) |  |  | 0.050370  (0.15586) | 0.054907  (0.15245) |
| Political view |  |  | -0.055123  (0.02834) | -0.06337  (0.02831) |  |  | 0.001976  (0.03004) | 0.008640  (0.02979) |
| Political view ∙ Non-student |  |  | 0.049807  (0.03403) | 0.057430  (0.03369) |  |  | 0.029054  (0.03929) | 0.016636 (0.03914) |
| Religiosity |  |  | 0.185383  (0.08513) | 0.194619  (0.08201) |  |  | -0.205167  (0.11687) | -0.22015  (0.12126) |
| Religiosity ∙ Non-student |  |  | -0.278169  (0.23805) | -0.31224  (0.24718) |  |  | 0.1475758  (0.0857) | 0.155358  (0.08191) |
| Net income |  |  | 0.216217  (0.10455) | 0.265238  (0.11651) |  |  | -0.151825  (0.08954) | -0.14411  (0.08877) |
| Net income ∙ Non-student |  |  | -0.203935  (0.10764) | -0.25163  (0.11974) |  |  | 0.147897  (0.0952) | 0.149215 (0.09447) |
| Trust |  |  | 0.005539  (0.06403) | 0.007230  (0.06308) |  |  | 0.1463999  (0.07218) | 0.116483  (0.07193) |
| Trust ∙ Non-student |  |  | -0.026415  (0.08552) | -0.02969  (0.08373) |  |  | -0.195071  (0.1071) | -0.18022  (0.10702) |
| Victim sensitivity |  |  | 0.096485  (0.04095) | 0.084583  (0.04082) |  |  | -0.054865  (0.04209) | -0.05001  (0.04235) |
| Victim sensitivity ∙ Non-student |  |  | -0.108535  (0.0536) | -0.09717  (0.05265) |  |  | 0.0866424  (0.06505) | 0.076556  (0.06531) |
| Beneficiary sensitivity |  |  | -0.029151  (0.03916) | -0.02237  (0.03845) |  |  | 0.0663425  (0.04183) | 0.073852  (0.04307) |
| Beneficiary sensitivity ∙ Non-student |  |  | 0.099412  (0.05549) | 0.090116  (0.05456) |  |  | -0.082997  (0.0625) | -0.09103  (0.06371) |
| Prob > chi2 | 0.8285 | 0.1090 | 0.0122 | 0.0161 | 0.0841 | 0.6217 | 0.2080 | 0.1481 |
| Pseudo R2 | 0.0046 | 0.0468 | 0.1988 | 0.2072 | 0.0342 | 0.0181 | 0.1335 | 0.1545 |

**Table A7 Regressions to explain trust behaviors**

| Logit (Marginal effects)  Y=1, trust message  Y=0, else | I | II | III | IV |
| --- | --- | --- | --- | --- |
| Non-student | -0.2380131  (0.24836) | -0.1511299  (0.1283) | 0.5801352  (0.60076) | 0.7097164  (0.58592) |
| Treatment 1 |  | -0.083808  (0.13967) | 0.1207992  (0.14739) | 0.2793095  (0.11782) |
| Treatment 1 ∙ Non-student |  | 0.0987528  (0.15842) | -0.1278271  (0.23238) | -0.4110783  (0.31076) |
| Treatment 2 |  | -0.2363331  (0.13529) | -0.1010371  (0.14772) | -0.1090308  (0.14928) |
| Treatment 2 ∙ Non-student |  | 0.0985407  (0.15227) | -0.0450665  (0.19466) | 0.0853431  (0.13657) |
| Expectation | 0.013703  (0.00303) |  | - | 0.0134817  (0.00323) |
| Expectation ∙ Non-student | 0.003214  (0.00498) |  | - | 0.0042527  (0.00574) |
| Age |  |  | 0.0016341  (0.016) | 0.0067358  (0.01589) |
| Age ∙ Non-student |  |  | -0.0056271  (0.01642) | -0.0114464  (0.01637) |
| Female |  |  | 0.0851633  (0.11397) | 0.0541548  (0.11369) |
| Female ∙ Non-student |  |  | -0.2561306  (0.24146) | -0.2285346  (0.41627) |
| Political view |  |  | -0.000155  (0.03725) | -0.0028053  (0.0355) |
| Political view ∙ Non-student |  |  | -0.0248402  (0.0453) | -0.0461145  (0.04621) |
| Religiosity |  |  | 0.2055029  (0.10664) | 0.1651204  (0.09528) |
| Religiosity ∙ Non-student |  |  | -0.3605198  (0.20651) | -0.23423  (0.26129) |
| Net income |  |  | -0.1419103  (0.11505) | -0.0430241  (0.10758) |
| Net income ∙ Non-student |  |  | 0.1595695  (0.1191) | 0.080314  (0.11378) |
| Trust |  |  | 0.2427153  (0.08243) | 0.1815842  (0.07338) |
| Trust ∙ Non-student |  |  | -0.1089127  (0.11168) | -0.1187129  (0.09972) |
| Victim sensitivity |  |  | -0.0241494  (0.05015) | -0.0372926  (0.04924) |
| Victim sensitivity ∙ Non-student |  |  | -0.0075174  (0.07159) | -0.0524449  (0.07866) |
| Beneficiary sensitivity |  |  | 0.0611266  (0.05465) | 0.0516786  (0.04686) |
| Beneficiary sensitivity ∙ Non-student |  |  | -0.0208508  (0.06978) | 0.0067163  (0.06531) |
| Prob > chi2 | 0.0000 | 0.2684 | 0.0233 | 0.0000 |
| Pseudo R2 | 0.3121 | 0.0283 | 0.1581 | 0.4436 |
