# Supplementary material for: Is there a link between endowment inequality and deception? – an analysis of students and chess players
Source: PLoS One. 2022 Jan 27;17(1):e0262144. doi: 10.1371/journal.pone.0262144 (PMC8794128; doi:10.1371/journal.pone.0262144)
Supplement: Attachment — Submitted filename: Response to Reviewers.docx [file pone.0262144.s005.docx]

**PONE-D-21-22531R1**

**Is there a link between endowment inequality and deception? – An analysis of students and chess players**

**PLOS ONE**

Many thanks for your comments. Please find attached our response along with the changes we have made in the manuscript.

**- You need to further develop the possibility of having different identities. It should be added to the conclusions as a potential explanation for different results. Regarding income how it was calculated should be explained.**

- Thanks for this point. We added the possible reason (different identities) to the Conclusion Section: “Reminding the subjects of their identity might be a driver for differences.”
- The variables are explained in Table 4. For example, we used several categories to collect data on net income: “less than €750 (=1), €750 up to less than €1,500 (=2), €1,500 up to less than €2,000 (=3), €2,000 up to less than €2,500 (=4), €2,500 up to less than €3,000 (=5), more than €3,000 (=6)”

**- Regarding order effects I think that this is a major drawback and as such you should mention it in the conclusions rather than in a footnote.**

We now say it not only in the experimental design (we think this should also be said there) but also in the Conclusion Section talking about limitations.

“Replication studies must show whether our findings are artifacts or systematical. For example, it is an open question whether order effects influenced our results.”

**- Even though you do not directly answer the hypothesis I think you should clarify your result section so that is easier for the reader to understand the main results obtained from your study.**

Yes, you are right. Results is a better description than “Data analysis”. Section 6 is not labeled as Results.

**Other comments**

**- I think that mentioning the main results in the introduction would add to the paper but I respect that you think otherwise.**

Thank you. Yes, there are different ways in the literature. ☺

**- Regarding the formula Fehr and Schmidt (1999) I would comment the purpose of having it there and that you are not using it further so that it is not misleading.**

We now introduce the formula as follows:

“For illustration purposes, this will be presented formally (although the formula will not be used later in the paper):”

**- I feel that you did not answer to my comment: “It may be the case that when receivers have a low endowment they do not trust senders because they may believe that they will try to even the payoffs”. I would add this as a possible explanation.**

We think that this point is somewhat related to the definition issue we have written in the paper. This might be the case that we did not consider it in the revision (lost in thoughts). This was also in the old version of the paper: “To keep it simple, we stick to the terminology of Gneezy (2005) in this paper. However, we would like to point out that this dichotomy (honest vs. deception) could also be seen critically. For example, as Sutter (2009) correctly points out honest messages could also be classified as deception if one has the expectation that the receiver does not follow the message.”

And now, we added the following sentence:

“It should also be mentioned that receivers, who have less initial endowment than their opponents, might not trust the senders because the former could believe that the latter may try to even the payoffs as a fairness criterion.”

**- It was difficult to understand this sentence of the introduction “Player 1 sends a message to player 2, which of the two options is supposedly financially advantageous for player 2. “ I think you cound add something like stating, explaining…after the comma.**

We think that we missed a word in our sentence and made it hard to read. We added “indicating” after the comma (but stating was also fine to us). Thank you.

**- Clarify in the introduction if the initial endowment is salient for all players.**

In the Introduction, we describe the basic design along with the treatment conditions where we systematically vary in the endowment:

“In our extended Gneezy (2005) design, we provide either player 1 or 2 with an initial endowment of €10 in the treatment conditions. In accordance with Fehr and Schmidt (1999), we distinguish between monetary advantageous inequality and monetary disadvantageous inequality.”
