# Supplementary material for: Is there a link between endowment inequality and deception? – an analysis of students and chess players
Source: PLoS One. 2022 Jan 27;17(1):e0262144. doi: 10.1371/journal.pone.0262144 (PMC8794128; doi:10.1371/journal.pone.0262144)
Supplement: Attachment — Submitted filename: Referee report PONE.pdf [file pone.0262144.s003.pdf]

Referee report PONE-D-21-22531: Is there a link between endowment inequality and deception? – An analysis of students and chess players

The goal of this paper is to investigate the relationship between inequality in endowment and deception experimentally. T do so; the authors selected Uri Gneezy's Deception game (Gneezy 2005). In this two-player game, player roles have very differentiated actions. While player 1 has private information regarding the payoffs of both players. The Player's one action could be to reveal the real payoffs or deceive the game. Player 2 has no information about payoffs but chooses between two actions that decide the payments of both parties. In this article, the authors vary the relative endowments of both players providing additional treatments where either Player, 1 or 2, have larger endowments. In addition to the experimental extension, the authors test the behavior with two different populations, students and chess players. Overall, the article addresses a very interesting issue, relating how inequalities could be a driver of deception. Besides this, I believe that the authors' sin was to be too ambitious. Thus, even when aiming to implement experiments with non-standard populations is a desirable goal of economic experiments, the authors were short at justifying why this particular population, chess players, are of particular interest when analyzing this relationship. Therefore, the design appears to be an ingenious experiment with two populations selected out of convenience to the reader. In the following paragraphs, I will provide a series of comments and suggestions to improve the manuscript.

From the abstract and the introduction of your article, the reader could easily identify two goals of the article: contribute to the understanding of the relationship between inequalities and deception and improve the external validity of the results in experimental economics. While I think that the experimental design provides us with one step forward to reaching our first goal, I cannot stop asking, "why should we particularly care about the deceptive behavior of chess players? This question was raised by Frechette (2011) in the discussion when he mentions:

"It also raises the question of what is the group of interest? Who are the agents that are supposed to be represented in those models being tested?"

Thus, how does the selection and understanding of the differences in chess players' behavior, in this case, improve the external validity of your results? I believe that you should develop on answering these questions in your introduction and extending your literature review on different samples of experimental subjects and the implications of observing differences in the behavior of these types in experimental games. Good examples to follow are one given by Abbink and Rochenbak (2006), Alevy et al. (2009), where they explain why understanding the behavior of financial advisors in a cascade game is crucial to improve our understanding of cascades.

Furthermore, the introduction of two types of subjects increases your hypothesis. Thus, you should provide power estimations that drive your experimental design and the choice of your number of observations per treatment and correct when needed for multiple hypothesis testing. I don't know if I failed to discover this analysis and values, but it is very important to perform these tasks even more when we have multiple populations.

While reading the article, I found some style and expression habits that I believe a difficult reader understands your work. For example, the article has several instances where the terms such as Player 1 and Player 2 are used to label tables. Then the reader finds that some other sentences are labeled as sender and receiver, which are used, for example, to name sub-sections of the paper leads the reader in a constant loop of mapping between several different terms to understand the text of your article. It would be an improvement in terms of the article's readability if you could reduce the diversity of terms or use them more systematically. N additional concept that appears to be misleading to any readers is your definition of "Market implications," this Is a two-person game with a context that Vernon Smith classifying inside the "personal exchange" (Smith 2009). There is no market because subjects are not exchanging a good or service, and there is no price or fee. I think that to be more traditional with the economist interpretation of the market and 2 by 2 experimental games; your article will benefit

I believe that the revision of related literature should be expanded, and the text should highlight how alternative theoretical frameworks and previous experiments related to your design and results. For example, little is said about how endowment inequalities affect behavior in other experimental games while there is ample evidence of endowment inequalities among others affecting behavior in trust games such as the one given by: Anderson et al. 2006, Ciriolo 2007, Lei & Vesely 2010, Xiao & Bicchieri 2010, Smith 2011, Brülhart & Usunier 2012, Hargreaves-Heap et al. 2013, Calabuig et al. 2016, Rodriguez-Lara 2018, Bejarano et al. 2018, 2021. Similarly, due to the lack of revision of different theoretical foundations of the relationship between inequality and deception, it appears that there is only one theoretical framework to base your hypothesis is only based on Fehr and Schimdt (1999). A set of new theories of deception Ettinger and Jehiel (2010) or even some interpretations of Mazar et al., 2008 could also inform you, the reader, and other researchers of how your hypothesis and results are in place within the larger literature on deception.

Finally, I consider that your statement regarding p-values and econometric analysis is understandable regarding the analysis. And even after carefully reading your footnote 4" where you stated: "we refrain from using asterisks and provide complete p-values instead, we reduce the number of econometric specifications than originally intended and pre-registered due to space restrictions. For a comprehensive overview of the specification, see the Appendix."

I cannot stop thinking that there is some inconsistency in your analytical approach. First, you stated reduced form regression expressions and present proportions and the outcomes of the statistical tests between proportions. Then you present tables with marginal effects of "only" one reduce form regression, the logit. But you do not talk about the robustness of the effects to different specifications or the need of clustering the errors, and you present the p-values in these tables. Thus this inconsistency claiming that p-values are not the only important factor and then presenting tables with p-values, but abstaining from defining a clear analytical criterion of analysis difficult readers comprehension of your results. Finally, I can not stop thinking that you do not have many significant results in statistical and non-statistical meaning. Worse than not having significant results is not clearly stating the results or lack of them. I think that the readers would appreciate it if you could clarify the criteria for your analysis. Even if you produce the traditional analysis, show the significance of these results and state your position within the body of the text in the results and discussion section in a more ordinated fashion.

Some References

Abbink, Klaus, and Bettina Rockenbach. "Option pricing by students and professional traders: a behavioral investigation." *Managerial and Decision Economics* 27.6 (2006): 497-510.

Alevy, J. E., Haigh, M. S. and List, J. A.: 2009, Information cascades: Evidence from a field experiment with financial market professionals, Journal of Finance 62(1), 151—80.

Ettinger, D. and Jehiel, P., 2010. A theory of deception. *American Economic Journal: Microeconomics*, *2*(1), pp.1-20.

Fréchette, G. R. (2011). Laboratory experiments: Professionals versus students. *Available at SSRN 1939219*.

Mazar, N., Amir, O. and Ariely, D., 2008. The dishonesty of honest people: A theory of self-concept maintenance. *Journal of marketing research*, *45*(6), pp.633-644.

Smith, V.L., 2009. Rationality in economics. *Cambridge Books*.
